# Late central graft detachment due to double endothelial layer after repeat Descemet membrane endothelial keratoplasty

**DOI:** 10.1016/j.ajoc.2023.101883

**Published:** 2023-07-03

**Authors:** Maximilian Friedrich, Hyeck-Soo Son, Ramin Khoramnia, Gerd Uwe Auffarth, Victor Aristide Augustin

**Affiliations:** Department of Ophthalmology, University Hospital Heidelberg, Im Neuenheimer Feld 400, 69120, Heidelberg, Germany

**Keywords:** Late graft detachment, Central detachment, Descemet membrane endothelial keratoplasty, Endothelial cell density, Cornea, Fuchs' endothelial corneal dystrophy

## Abstract

**Purpose:**

To report late central graft detachment after repeat Descemet membrane endothelial keratoplasty (DMEK) without visual reduction.

**Observations:**

A 71-year-old patient with Fuchs’ endothelial corneal dystrophy received a DMEK in his left eye. At 11 month post-operatively, a subtotal graft detachment was noted. Due to increasing corneal edema with vision loss, the first DMEK was removed and a repeat-DMEK was performed. At four months post repeat-DMEK, the graft was fully adherent to the posterior stroma. There was no significant corneal edema, and the best corrected visual acuity was 20/25. At 16-months after repeat-DMEK, a central graft detachment was noted, but there was no concurrent corneal edema or any loss of visual acuity. The mean density of the central endothelial cells was measured at 842 cells/mm^2^. Given the lack of corneal edema, visual reduction or subjective visual complaint, the graft detachment was followed-up for up to 20-months post repeat-DMEK with no further intervention, where the central cornea remained clear.

**Conclusions and Importance:**

To our knowledge, this is the first report of a central repeat-DMEK graft detachment that occurred 16 months after surgery despite initial attachment. Interestingly, there was no concurrent corneal edema or vision reduction. We describe a potential mechanism for clear central cornea in the presence of a central graft detachment after repeat-DMEK.

## Highlights

• Isolated central graft detachment can occur after repeat-DMEK.
• Central graft detachment can occur without loss of visual acuity or corneal edema.
• Endothelial cell density should be measured before repeat-DMEK.
• Stromal polishing may be performed during repeat-DMEK before graft insertion.
• Central graft detachment with good visual acuity after repeat-DMEK can be tolerated.

## 1 Introduction

In patients with Fuchs’ endothelial corneal dystrophy (FECD), a progressive reduction of endothelial cell density leads to inadequate dehydration of corneal stroma, ultimately causing corneal edema and visual impairment.^1^ Descemet membrane endothelial keratoplasty (DMEK) is currently the treatment of choice for FECD patients. By replacing the diseased endothelium with a functioning endothelial layer, the fluid transport is reconstituted, and the corneal clarity can be restored.^2^^,^^3^

Despite high success rates and good visual outcomes reported after DMEK, postoperative success can be compromised by a number of complications.^4^^,^^5^ In particular, previous studies have reported peripheral graft detachment to occur in 26–56% of cases.^6^^,^^7^ Without further treatment, there is a high risk of vision loss as it may progress to detach completely. Thus, in cases of peripheral or complete graft detachment, a re-bubbling or repeat-DMEK may need to be performed.^7^

Graft detachments are typically observed in the early postoperative period, which highlights the importance of close follow-up examinations following the surgery. To our knowledge, a graft detachment occurring numerous months after surgery has not been reported before, particularly a detachment that *only* involves the graft center. This case report presents a case of a *late* central graft detachment occurring after repeat-DMEK in an eye that previously underwent DMEK for FECD.

## 2 Case report

A 71-year-old White male presented to our department with blurry vision in his left eye. He had previously undergone DMEK in this eye 11 months ago for corneal decompensation due to FECD. Other previous ocular history was unremarkable except for cataract surgery on both eyes more than 10 years ago. His systemic comorbidities included myasthenia gravis, rheumatoid arthritis, and obstructive sleep apnea syndrome.

**Fig. 1:**
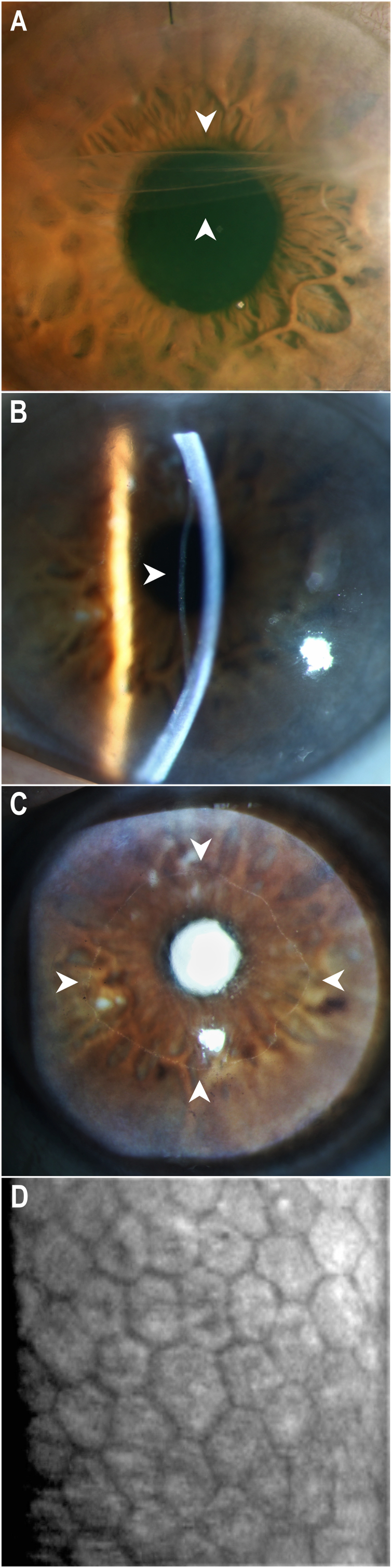
Slit lamp examination of the left eye one day before and 20 months after Descemet membrane endothelial keratoplasty (DMEK). **A**: Subtotal graft detachment of the first DMEK graft is visible one day before repeat-DMEK. **B**: Central graft detachment is observable due to the concave light reflex in endothelial focus 20 months after the repeat-DMEK. **C**: Using a broad light beam the border of the central graft detachment is visible 20 months after the repeat-DMEK. **D**: Imaging of the central endothelial cells on the posterior stroma with a specular microscope.

The left eye showed a best-corrected visual acuity (BCVA) of 20/200, and the slit-lamp examination revealed a diffuse corneal edema involving the optical axis. Optical coherence tomography (OCT) demonstrated a subtotal DMEK graft detachment (Fig. 1A) and a central corneal thickness of 552 μm. Given the clinically significant corneal decompensation in the setting of a subtotal graft detachment, the patient was advised to undergo repeat DMEK.

**Fig. 2:**
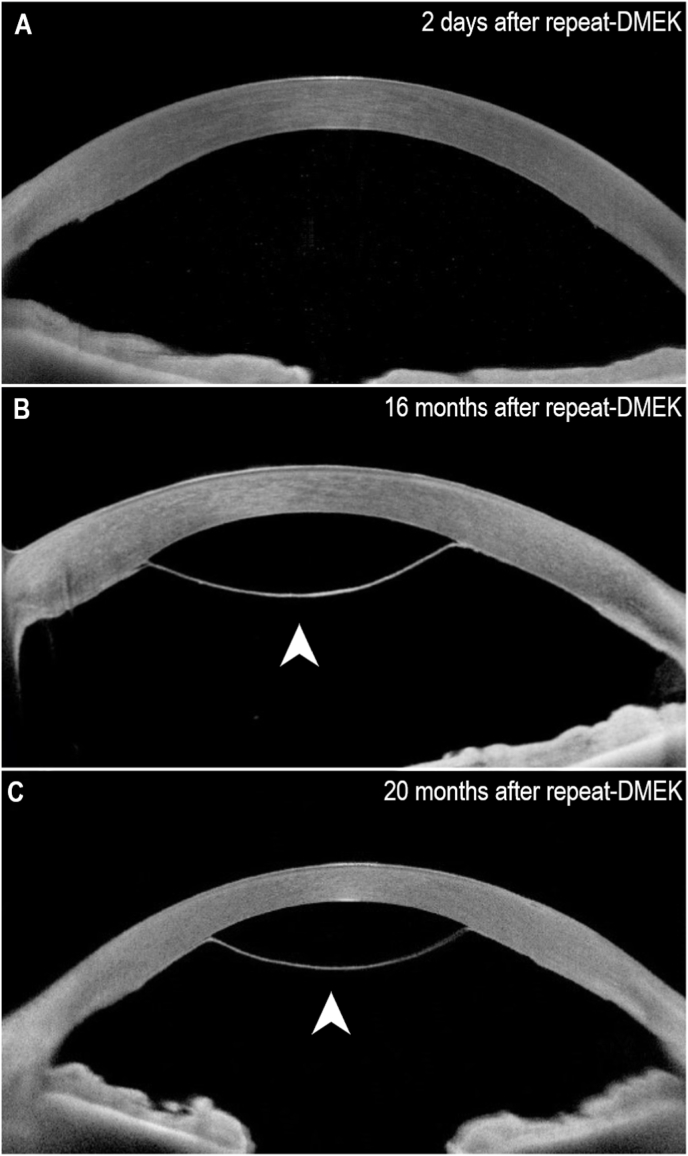
Development of central graft detachment in optical coherence tomography. **A:** 2 days after the repeat Descemet membrane endothelial keratoplasty (DMEK) the graft is fully attached to the corneal stroma. **B:** 16 months after the repeat-DMEK central graft detachment is observable. **C:** 20 months after the repeat-DMEK the central graft detachment remains constant.

**Fig. 3:**
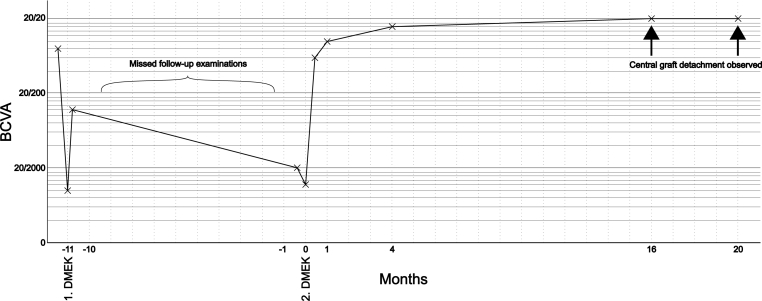
Best corrected visual acuity after Descemet membrane endothelial keratoplasty (DMEK). Despite central graft detachment between 4 and 16 months after the repeat-DMEK, visual acuity was not compromised. BCVA = Best corrected visual acuity.

The repeat-DMEK was performed with no intraoperative complications and the new graft was completely attached to the stromal surface under 20% SF6 gas tamponade. Both the slit-lamp and the anterior-segment OCT confirmed a fully adherent graft position (Fig. 2A) at 2-days postoperatively. As shown in Fig. 3, the BCVA improved to 20/60 at 8-days postoperatively.

At 1- and 4 months after surgery, the graft was still fully attached to the host stroma and the BCVA had improved to 20/25. The central corneal thickness was 489 μm.

When the patient followed up at 16-months postoperatively, however, a central graft detachment was observed (Fig. 1B). A broad slit-lamp beam demonstrated a detached area of approximately 4.5 mm only in the central cornea (Fig. 1C). The BCVA was 20/20. There was no clinical corneal edema or corneal scarring. The anterior-segment OCT confirmed the central graft detachment with peripheral graft still fully attached to the corneal stroma (Fig. 2B). Central corneal thickness was 499 μm. The height of graft detachment was measured at 1.2 mm.

Given the good BCVA and the lack of any subjective visual complaints, we decided against a surgical intervention and followed-up in 4 months. At 20-months after repeat-DMEK, the magnitude of central graft detachment remained stable with consistent diameter and height of the detachment (Fig. 2C), and there were still no signs of corneal edema. The BCVA also remained at 20/20 and the central corneal thickness was 487 μm. A specular microscope (CEM-530, NIDEK, Gamagori, Aichi, Japan) was used to measure the endothelial cell density in the central cornea, which showed a mean endothelial cell count of 841.7 ± 57.6 cells/mm^2^. The HRT3 Rostock Cornea Module (Heidelberg Engineering, Heidelberg, Germany), which is widely used to analyze the depth and composition of corneal layers, confirmed the location of the endothelial cells in the central posterior corneal stroma. Given the clinically stable situation with no visual impairment, we decided to continue observation with no further surgical intervention.

## 3 Discussion

DMEK graft detachment generally occurs in the early postoperative period and is often accompanied by vision loss due to increasing corneal edema in the area of detachment.^8^^,^^9^ In our case, however, the repeat-DMEK graft not only detached more than 3 months after surgery, but also *only* occurred in the central cornea with no signs of concurrent corneal edema or loss of visual acuity.

It is unclear which exact mechanism led to late graft detachment that only involves the central cornea. In our case, a subtotal graft detachment was first observed at 11-months after the initial DMEK. As the patient had failed to follow-up between 1 and 11 months after the initial DMEK, it is unknown for how long the graft had remained detached in this eye as shown in Fig. 1A. However, there seems to have been a physical contact between the endothelial side of the detached graft tissue and the central posterior stroma, leading us to hypothesize that this may have served as a “bridge” for endothelial cells to slowly migrate onto the central posterior stroma. Thus, the repeat-DMEK graft was most likely transplanted onto the migrated endothelial cells of the first graft on central posterior stroma.

Immediately after repeat-DMEK, the migrated endothelial cells on central posterior stroma, together with peripheral endothelial cells from the second DMEK graft, then presumably began to dehydrate the edematous cornea. This is reflected in the gradual improvement of BCVA and corneal clarity at 4-months post repeat-DMEK, which is in alignment with a report by Lazaridis et al. who observed a recovery of corneal clarity at 3-months following DMEK.^10^ During this process, the stromal fluid transported by the migrated endothelial cells on central posterior stroma likely began to "push” the second DMEK graft away, leading to central detachment of the graft and progressive fluid accumulation between the endothelial cells and the second DMEK graft.

**Fig. 4:**
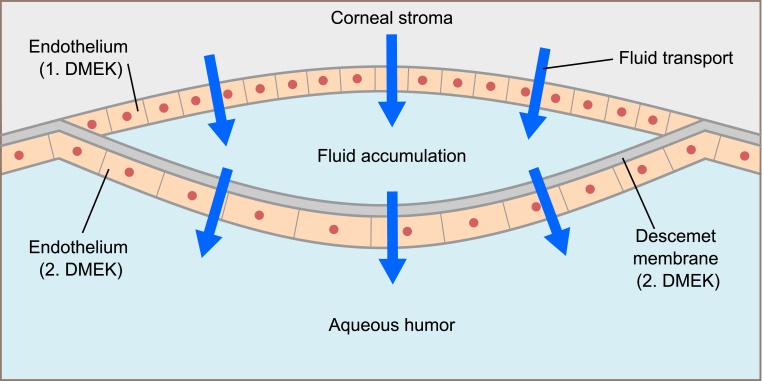
Illustration of the hypothetical process resulting in late central graft detachment. The corneal stroma is dehydrated via the fluid transport of the migrated endothelium of the first Descemet membrane endothelial keratoplasty (DMEK). The fluid accumulates between the migrated endothelium and the graft of the repeat-DMEK. The endothelium of the repeat-DMEK transports the fluid out of the accumulation into the aqueous humor of the anterior chamber.

In such a case, one would expect the gradual fluid “pouch” above the area of graft detachment to continue to increase and ultimately cause a total graft detachment. Interestingly, however, the size of graft detachment remained unaltered. One possible mechanism may be that the healthy endothelial cells residing in central repeat-DMEK graft also transport a similar amount of fluid from the accumulating fluid pouch, thereby establishing a certain balance in the amount of fluid that is being transported 1) from the central corneal stroma into fluid pouch and 2) from the fluid pouch into the anterior chamber (Fig. 4).

Surely, a re-bubbling may be a possible option to treat such a graft detachment. However, a re-bubbling in the setting of such a central graft detachment could apply pressure on the central fluid accumulation and lead to a further increase in graft detachment. A third DMEK may also be a possibility. However, the central corneal clarity as well as the lack of visual loss or complaints dissuaded us to perform a third corneal transplantation.

In conclusion, late central graft detachment can occur after repeat-DMEK, possibly due to endothelial cells that migrate from the first DMEK tissue onto the host posterior stroma. This suggests that stromal polishing may be useful when performing a repeat endothelial keratoplasty to remove all residual migrated endothelial cells. Central graft detachments with no associated corneal edema or visual loss can be observed with no surgical intervention. Future studies are necessary to confirm the mechanism of late repeat-DMEK graft detachment described herein.

## Patient consent

Written informed consent for publishing personal information and case details was obtained from the patient. A copy of the written consent is available upon reasonable request.

## Funding

No funding or grant support.

## CRediT authorship contribution statement

**Maximilian Friedrich:** Methodology, Investigation, Data curation, Writing – original draft, Visualization. **Hyeck-Soo Son:** Methodology, Data curation, Writing – review & editing, Visualization. **Ramin Khoramnia:** Resources, Writing – review & editing, Supervision. **Gerd Uwe Auffarth:** Resources, Writing – review & editing, Supervision. **Victor Aristide Augustin:** Conceptualization, Methodology, Investigation, Resources, Writing – review & editing, Supervision, Project administration.

## Declaration of competing interest

The authors declare the following financial interests/personal relationships which may be considered as potential competing interests: Ramin Khoramnia – Heidelberg Engineering – Lecture fee

The following authors have no financial disclosures:

M.F.; H.-S.S.; G.U.A.; V.A.A.
